# Racial residential segregation and child mortality in the southern United States at the turn of the 20th century

**DOI:** 10.1002/psp.2678

**Published:** 2023-06-07

**Authors:** J’Mag Karbeah, J. David Hacker

**Affiliations:** 1 Minnesota Population Center, University of Minnesota School of Public Health, Minneapolis, Minnesota, USA; 2 Department of History, Minnesota Population Center, University of Minnesota, Minneapolis, Minnesota, USA

**Keywords:** child mortality, historical demography, IPUMS, race disparities, segregation

## Abstract

A growing body of research considers racial residential segregation to be a form of systemic racism and a fundamental cause of persistent racial disparities in health and mortality. Historical research examining the impact of segregation on health and mortality, however, is limited to a few studies with poor data and inconsistent results. In this study, we examine the association between racial residential segregation and child mortality in the South at the turn of the 20th century. We rely on the new IPUMS 1900 and 1910 complete-count databases to estimate child mortality in the 5 years before each census and construct segregation measures at the census enumeration district (ED), the lowest level of geography consistently available in the census. We calculate the proportion of households headed by Black individuals in each ED, and the Sequence Index of Segregation (*SIS*), which is based on the racial sequencing of household heads within each district. We construct models of child mortality for rural and urban areas, controlling for a wide variety of demographic and socioeconomic variables. The results indicate that *proportion Black* and *SIS* were strongly and positively associated with the mortality of Black children in most models and in both rural and urban areas. *Proportion Black* was also positively but more moderately correlated with the mortality of White children, while *SIS* was not correlated or negatively correlated. These results suggest that racial segregation was a long-standing fundamental cause of race disparities in health and mortality in the United States.

Racial residential segregation has been described as a fundamental cause of race disparities in health and mortality, the ‘cornerstone’, according to David R. Williams and Chiquita Collins, ‘on which Black–White disparities in health status has been built in the US’ (Williams & Collins, 2001). Segregation results in the clustering of socioeconomic disadvantage within minority communities, whose residents are exposed to greater environmental dangers and crime, live in lower quality housing, and experience limited access to high-quality education, health care, and labour markets (Acevedo-Garcia & Osypuk, 2008; Almond et al., 2006; Cutler & Glaeser, 1997; Cutler et al., 1999; Massey & Denton, 1993; Williams & Collins, 2001). Researchers have documented significant relationships between This is an open access article under the terms of the Creative Commons Attribution License, which permits use, distribution and reproduction in any medium, provided the original work is properly cited. segregation and health across the life course, including disparate outcomes in perinatal measures, birth weight, infant and child mortality, childhood asthma, teen pregnancy, obesity, diabetes, hypertension, and health behaviours in adulthood (Acevedo-Garcia et al., 2007; LaVeist et al., 2011; McFarland & Smith, 2011; Mehra et al., 2017; Mendez et al., 2014; Osypuk et al., 2009). Although causal pathways linking residential segregation to health and mortality are likely multiple, vary across different contexts, and are often unclear and difficult to identify, a growing body of research considers segregation to be a form of systemic racism that contributes to persistent and fundamental disparities in socioeconomic status and health outcomes (Feagin & Bennefield, 2014; Owens & Fett, 2019; Phelan & Link, 2015).

Contemporary patterns of racial residential segregation in the United States are the result of historical practices and norms originating in slavery (1619–1865) and modified during Reconstruction (1865–1877), the Jim Crow era (beginning c. 1877), the ‘Great Migration’ of millions of rural southern Black individuals to northern cities (c. 1915–1970), and ‘White flight’ from central cities to suburbs after the second world war (Boustan, 2010; Clark, 1986; Glaeser & Vigdor, 2012; Gregory, 2005; Leibbrand et al., 2020; Massey & Denton, 1993; Rothstein, 2017; Ruef, 2022; Wright et al., 2014). This history has potential relevance for understanding race disparities in mortality in the past and the persistence of disparities from the 19th century to the present (Jackman & Shauman, 2019; Singh & Yu, 2016). An important unanswered question is whether the relationship between residential segregation and mortality developed only in the late 20th century or whether the relationship was present over a much longer span of time. In other words, is racial residential segregation a *fundamental* cause of race differences in mortality (Phelan & Link, 2015; Williams & Collins, 2001) or is the relationship a relatively new phenomenon applicable only in modern contexts? Recent research indicating that greater exposures to segregated and high-poverty neighbourhoods in childhood can have long-lasting effects on educational attainment, earnings, and health in adulthood (Chetty et al., 2016), that neighbourhood inequality is mutigenerational (Sharkey, 2013), and that ‘redlining’ practices in the early 20th century (the racially discriminatory grading of neighbourhoods’ credit worthiness) are associated with present-day health inequalities (Krieger et al., 2020) are additional reasons to investigate historical patterns of segregation. Historical research examining the association between residential segregation, health, and mortality, however, is limited to a few studies with poor data and inconsistent results (e.g., Logan & Parman, 2018; Yankauer, 1950). As a result, considerable uncertainty remains about the strength and even the direction of the relationship between segregation and race differentials in health and mortality before the late 20th century.

This study examines the relationship between racial residential segregation and child mortality at the turn of the 20th century using newly-constructed IPUMS complete-count microdata databases of the 1900 and 1910 censuses (Ruggles et al., 2021). The IPUMS datasets illustrate the enormous potential for using historical census data to test hypotheses: the combined datasets include data on every individual enumerated in the two censuses, some 168 million cases combined, with diverse backgrounds, occupations, family structures, and residence characteristics. The datasets are complete transcriptions of the census returns with each individual’s age, sex, race, birthplace, marital status, occupation, literacy, homeownership, recent unemployment, relationship to the head of household, place of residence, and much more information that can be measured directly or inferred, such as the characteristics of nearby neighbours. Both the 1900 and 1910 censuses included information on the number of women’s children ever born and the number of those surviving at the time of the census, which can be used to measure child mortality. Although the datasets have a few shortcomings, which are described in more detail below, they allow examination of the relationship between racial residential segregation and child mortality in a period well before existing studies.

We measure racial residential segregation at the lowest level of aggregation available in the data—the census enumeration district (ED) level, which typically conformed to recognised neighbourhoods and contained an average of about 2000 residents. We construct two segregation measures, the proportion of all households in the ED headed by Black individuals (*proportion Black*), and the Sequence Index of Segregation (*SIS*), a more fine-grained measure of segregation based on the sequential race patterning of household heads (Grigoryeva & Ruef, 2015). We compare the relationship between racial residential segregation and child mortality in rural areas, where a large majority of the Black population at the turn of the 20th century lived, and in urban areas. We interact *proportion Black* and *SIS* with children’s race in all models to determine whether segregation had a different relationship with the mortality of Black and White children.

Because 89.9% of the Black population in 1900 resided in the South, we confine our analysis to the Black and White populations living in the 16 states and the District of Columbia that composed southern census regions.^1^ Despite that restriction, the two datasets allow us to estimate the association between segregation and the mortality of 4.7 million Black and White children aged 0–4 while controlling for a wide range of demographic, socioeconomic, and residential variables.

Given our lack of prior knowledge about the relationship between mortality and racial residential segregation in the past and inherent drawbacks of cross-sectional census data, which do not lend themselves to the estimation of causal relationships, our analysis is exploratory and research questions are largely descriptive. Was racial residential segregation correlated with child mortality at the turn of the 20th century? If so, did the relationship vary by children’s race, type of segregation, and between urban and rural populations? Finally, what does the relationship between racial residential segregation and child mortality at the turn of the 20th century suggest about segregation as a fundamental cause of long-standing inequities in health and mortality?

## 1 | BACKGROUND AND PRIOR STUDIES

Residential segregation is the spatial separation of population groups within a specified geographic area. Groups can be defined by ascribed characteristics such as race, foreign birth, and ethnicity or achieved characteristics such as religion, income, and education. Residential segregation is typically theorised to be harmful for minority populations and groups with low socioeconomic status and beneficial for majority populations and groups with high socioeconomic status, primarily through neighbourhood effects of concentrated poverty or affluence, but also along a variety of other possible causal pathways such as discriminatory practices independent of economic factors and psychosocial factors (Krieger, 2014; Timberlake & Ignatov, 2014). In the United States, research has focused on racial residential segregation and its impact on socioeconomic status and other measures of wellbeing (e.g., LaVeist, 1993; Massey & Denton, 1993), while in Europe it has focused more on ethnic, religious, and class segregation (e.g., Connor, 2017; Lloyd & Shuttleworth, 2012; Musterd, 2005; Musterd et al., 2017). Some research suggests that residential segregation may be beneficial for minority populations along some dimensions of wellbeing and in certain contexts. Higher-density minority populations, for example, has been found to reduce the amount of racism experienced by minorities in England, resulting in improvements in mental health (Becares et al., 2009), and philosophical and empirical arguments have been made in defence of ‘voluntary separation’ (Merry, 2013). The temporal context may matter as well. Collins and Margo (2000) found that racial segregation in urban areas of the United States was negatively correlated with socioeconomic outcomes for the Black population only after 1970. Before that date, they speculated that racial residential segregation required most Black people to conduct transactions in racially segregated markets, which increased the demand for the services of black-owned businesses and for black labour.

Research on residential segregation has been interdisciplinary, with important contributions from economists, sociologists, geographers, demographers, social epidemiologists, and historians. Most published research on the relationship between racial residential segregation and population health, however, has been conducted by sociologists and public health researchers, who have focused on identifying neighbourhood effects and the health impacts of racism and discrimination on a variety of health outcomes (Acevedo-Garcia et al., 2003; Collins & Williams, 1999; Kramer & Hogue, 2009; Mehra et al., 2017; Williams & Collins, 2001). In the United States, racial residential segregation is frequently conceptualised as an example of ‘structural racism’—the totality of ways in which racial discrimination is ‘deeply embedded in systems, laws, written or unwritten policies, and entrenched practices and beliefs that produce, condone, and perpetuate widespread unfair treatment and oppression of people of colour, with adverse health consequences’ (Braveman et al., 2022). Residential segregation has been shown to have a negative impact on Black population health across the life course, independent of other risk factors (Mehra et al., 2017; Williams & Collins, 2001) and has occasionally been shown to be beneficial to white population health (Beaulieu & Continelli, 2011; Massey, 2001, p. 338). Several pathways have been identified linking racial residential segregation and racial differences in mortality and morbidity, including the role of residential segregation in limiting Black individuals’ access to protective resources such as adequate health care, limiting access to safe and well-paid occupations, increasing stress and related psychosocial factors, and increasing exposures to risk factors associated with concentrated poverty, poor housing, higher crime rates, and toxic environments. White individuals, in contrast, benefit from living in areas with more concentrated affluence, lower crime rates, higher quality housing, better health care, and better environments. These differential exposures have both acute and chronic consequences and have been correlated with adverse later-life outcomes for Black individuals such as increased risk of chronic disease. There are likely many potential causal pathways connecting residential segregation specifically and structural racism more generally to health disparities, however, which have proven difficult or impossible for researchers to identify precisely (Bailey et al., 2017; Gee & Ford, 2011; Williams & Mohammed, 2013).

Some of the factors identified with racial residential segregation in modern populations (e.g., concentrated poverty, poorer housing, and poorer access to high-quality education and jobs in Black neighbourhoods) were also likely relevant to the relationship between residential segregation and racial disparities in mortality in the early 20th century. Compared to modern settings, however, the historical context in the early 20th century United States was characterised by much different social, economic, environmental, and epidemiological conditions. Perhaps most importantly, causes of death and age patterns of mortality differed dramatically. In 2020, the infant mortality rate in the United States was 5.4 per 1000 births and degenerative diseases such as heart disease and cancer were the primary killers of individuals (Arias & Xu, 2022). In 1900, the infant mortality rate was 124.5 per 1000 and infectious diseases—spread by insect vectors, direct contact, and a variety of airborne and waterborne pathogens—were the leading causes of death (Glover, 1921; Grob, 2002). These contextual differences suggest a much greater role of residential segregation in past populations with respect to contagion than in modern populations. It was a period, according to demographic historian Daniel Scott Smith (1983), when ‘one was killed, so to speak, by his or her neighbours’. Among infants and young children, access to uncontaminated water, milk, and food also varied greatly across localities and neighbourhoods, highlighting the possible relationship between residential segregation, the salubrity of the local environment, and the spatial distribution of public health measures, including water supply and sewage systems (Preston & Haines, 1991).

**FIGURE 1:**
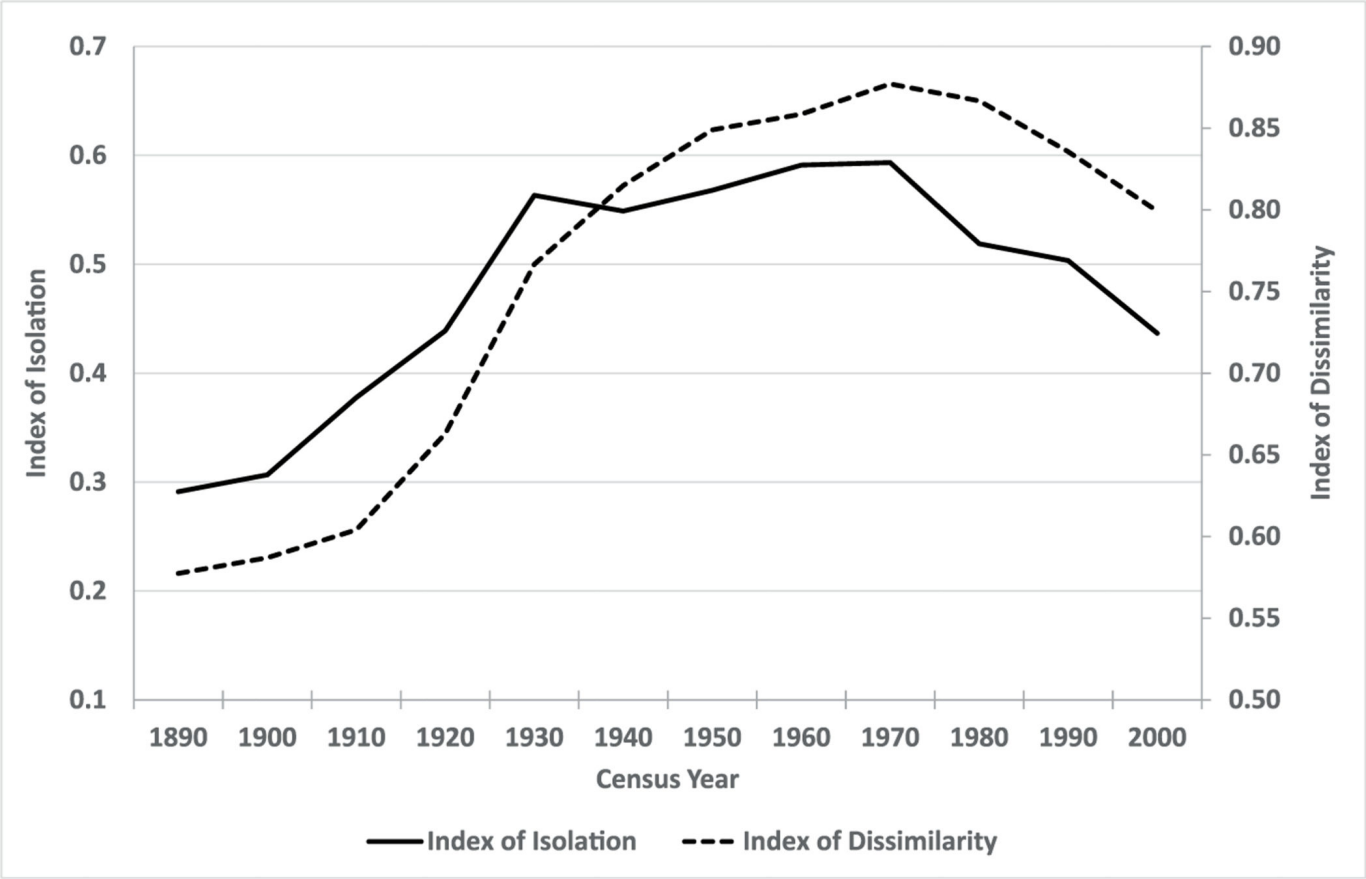
Racial residential segregation in the largest 10 U.S. cities, 1890–2000. *Source*: Shertzer et al. (2016).

Levels and patterns of segregation were also much different in the early 20th century United States than they are today. According to conventional measures of racial evenness and isolation, residential segregation was significantly lower in late 19th century U.S. cities than it has been since (Glaeser & Vigdor, 2012). These relatively low levels of segregation have been traced to residential patterns during slavery, when owners needed to live in close proximity to their slaves to ensure their supervision and control (Logan et al., 2015; Ruef, 2022; Woodward, 1955). Hanchett (1989), for example, emphasised the ‘salt-and-pepper’ racial pattern of residence in Charlotte, North Carolina where ‘African-Americans continued to live all over the city, usually side-by-side with Whites’ after the war. Blassingame (1973) observed similar patterns in Savannah, Georgia. Despite the beginnings of a discernable trend toward higher levels of racial residential segregation in urban areas during the Jim Crow era (beginning c. 1877), when policies requiring the social and physical separation of Black and White people were increasingly enacted and enforced, conventional segregation indexes indicate that Blacks in southern cities in 1910 were less segregated than newly arrived immigrants in northern cities (Glaeser & Vigdor, 2012; Massey & Denton, 1993; White et al., 1994). Racial residential segregation, however, accelerated during the ‘Great Migration’ (c. 1915–1970), when millions of Black people relocated from the rural South to urban cities in the North and West (Blassingame, 1973; Hanchett, 1989; Woodward, 1955), and with the legal imposition of discriminatory local, state, and federal housing and mortgage policies that became increasingly common in the half century before the Civil Rights movement in the 1960s (Rothstein, 2017). Nationally, as shown in Figure 1 for the top 10 cities in the United States, racial segregation increased each census year from 1890 to 1970, after which it declined to levels more characteristic of the early 20th century (Cutler et al., 1999; Glaeser & Vigdor, 2012; Leibbrand et al., 2020; Logan et al., 2011; Logan & Parman, 2017; Shertzer et al., 2016).

Although Black and White residents at the turn of the 20th century could be found in significant numbers in most neighbourhoods across southern cities, recent scholarship has emphasised that there was significant segregation *within* urban neighbourhoods. Black residents often resided in lower-quality and crowded housing on back alleys and smaller side streets while White residents resided in higher-quality housing fronting main streets (Grigoryeva & Ruef, 2015; Logan, 2017). Thus, significant residential segregation can be hidden in conventional segregation indexes such as the dissimilarity and isolation indexes, which summarise the overall level of segregation in a city or municipal area. Racial residential segregation in rural areas has received much less scholarly attention. A recent study by Logan and Parman (2017), however, found similar patterns and trends in racial residential segregation in rural areas of the South, with significant segregation within rural counties and a significant increase in the level of segregation between 1880 and 1940 paralleling increases in urban segregation. Examination of rural segregation patterns in rural areas is essential for the study of segregation in the early 20th-century South for the simple reason that approximately 9-in-10 Black individuals in 1900 and 1910 resided in rural areas.

Despite scholarly interest in long-term trends in residential segregation, demographic historians have largely ignored its potential role in race differentials in mortality. It is well known, of course, that Black and immigrant populations in the post-Civil War era suffered high rates of mortality from waterborne enteric diseases and from tuberculosis and other respiratory diseases associated with overcrowded and poorly ventilated housing, especially in urban areas (Ager et al., 2020; Ewbank, 1987; Feigenbaum et al., 2019; Roberts, 2009; Zelner et al., 2017). A recent county-level analysis of the early 20th-century South (Elman et al., 2019) found higher Black child mortality rates in counties with plantation agriculture, poor sanitation, and high overall mortality rates from malaria. Little systematic effort, however, has been made to estimate segregation’s relationship with racial disparities in mortality.

The lack of study likely reflects several factors, including the lack of high-quality mortality and segregation data and perceived low levels of racial residential segregation before the Great Migration. The relatively even distribution of Black and White residents across southern cities in the early 20th century likely made the practice of some types of place-based discrimination more difficult (Anderson et al., 2021; Troesken, 2004). Werner Troesken (2004), for example, has shown that public investments in water and sewer systems, which were increasingly made by southern cities in the late 19th and early 20th centuries, benefitted both Black and White residents, and that Black life expectancy in the early 20th century increased relative to White life expectancy. Chlorination of water supplies in the early 20th century also played a role in reducing the Black–White infant mortality gap (Anderson et al., 2021). In this context, racial residential segregation may not have been a significant factor in race differentials in mortality. In addition, as discussed above, there is some evidence to suggest that segregation may have benefitted Black-owned businesses and Black labourers before the mid 20th century via racially segregated markets (Collins & Margo, 2000).

Perhaps most importantly, prior research on the relationship between segregation and mortality was hampered by the limited availability of mortality data, especially at the individual and household levels. When the nation’s death registration system was first established in 1901, the Death Registration Area included just 10 states, none of which was in the South, and the District of Columbia (Ewbank, 1987; Preston & Haines, 1991). The use of vital registration data is complicated further by race differentials in the under-registration of deaths and the difficulty of linking these data to censuses with socioeconomic and residential information. As a result, most historical analyses of race differentials in mortality have been conducted with aggregate registration data (e.g., Elman et al., 2019; Eriksson et al., 2018; Feigenbaum et al., 2019; Yankauer, 1950), making it difficult or impossible to estimate the association between residential segregation patterns and mortality while controlling for socioeconomic status and other factors. Yankauer’s pioneering study of infant mortality in 318 residential areas in New York City (1950), for example, found that neighbourhoods with higher percentages of non-White births had higher infant mortality rates (for both Black and White children), but made no direct attempt to control for socioeconomic status. A few studies have relied on the number of children ever born and number of children surviving reported by mothers in the 1900 and 1910 censuses to estimate child mortality and its correlates at the individual and household levels (Dribe et al., 2020; Logan & Parman, 2018; Preston & Haines, 1991; Preston et al., 1994). Unfortunately, early researchers lacked access to complete-count or high sample density microdata, limiting analytical possibilities with respect to residential segregation.

Trevon Logan and John Parman’s recent exploratory analysis (Logan & Parman, 2018) of the relationship between county-level segregation in 1880 and mortality is the only published study to investigate the relationship between racial residential segregation and mortality before the Great Migration. Unfortunately, at the time of its research and publication, complete-count census microdata, which were needed to construct Logan and Parman’s neighbour-based county-level measure of segregation, were available only for the 1880 and 1940 censuses. Neither of these censuses included information on mortality, forcing the researchers to obtain data from other sources. Child mortality data were obtained from the 5% IPUMS sample of the 1900 census and the 1% IPUMS sample of the 1910 census (Ruggles et al., 2020) and linked to corresponding counties in the 1880 census. The results suggested that there was no relationship between segregation in 1880 and whether mothers had experienced the death of one or more of her children in 1900 or 1910, regardless of whether the mother was Black or White or lived in in an urban or rural county.

Logan and Parman also examined the correlation between racial residential segregation and adult age at death using digitised death certificates for adults born in North Carolina and dying in the state between 1909 and 1975. Racial residential segregation at the county level in 1880 was positively correlated with the age at death of Black males and females and negatively correlated with the age at death of White males and females born in rural areas. The relationship was strongly positive for Black males and females born in North Carolina counties defined as urban in 1880, suggesting that segregation was especially beneficial for urban Blacks. This result, however, was based on fewer than 2750 Black individuals born in just three North Carolina ‘urban’ counties, defined by Logan and Parman as counties with 20% or more of its households living in urban areas. Temporal mismatches between the measurement of segregation and the age at death of decedents (some deaths occurred as late as 1975, nearly a century after the county’s segregation level was measured in 1880), unobserved migration within the state, and unobserved migration out of the state—which was likely substantial during the Great Migration—are likely significant sources of bias, undermining confidence in these results.^2^ The use of more robust data, like complete-count IPUMS datasets, allows for a more detailed analysis of this potential relationship.

## 2 | DATA AND MEASURES

We rely on new IPUMS complete-count datasets of the 1900 and 1910 censuses (Ruggles et al., 2021) to measure child mortality, segregation, and other suspected covariates of mortality. The 1900 dataset contains over 76 million individual-level records, while the 1910 dataset contains over 92 million records. We used the public-use versions of these datasets, which are anonymous, meaning that individuals’ names are not included. Unique among U.S. censuses, the 1900 and 1910 censuses also asked each ever-married woman the number of living children she had given birth to and the number of those children who were still living at the time of the census. These data can be used to calculate the number of each mother’s children dying before the census, the number of her living children who were coresident in the household at the time of the census, and the number of her living children who were non-coresident at the time of the census. All public use IPUMS datasets can be freely downloaded from the Minnesota Population Center (https://ipums.org). Similar census datasets with children ever born and children surviving data are available for the 1911 censuses of Ireland and England and Wales and have been used to conduct studies of child mortality (e.g., Connor, 2017; Reid et al., 2016).

### 2.1 | Measurement of child mortality

We considered several measures of child mortality used by other investigators, including whether a mother had experienced the death of one or more of her children (Logan & Parman, 2018), the proportion of a mother’s children who were deceased at the time of the census, and a child mortality index standardised to a model life table (Connor, 2017; Dribe et al., 2020; Hacker & Haines, 2005; Haines & Preston, 1997; Preston et al., 1994; Preston & Haines, 1991; Reid et al., 2016). To minimise potential biases from unobserved migration and changes in other time-dependent variables, we took the more novel approach of reconstructing women’s complete birth histories (Luther & Cho, 1988) and basing our analysis on children born in the 5 years before each census. Reconstructed birth histories include the age of each child at the time of the census, their associated year of birth, the mother’s corresponding age at birth, and child’s birth order, regardless of whether the he or she was living or deceased at the time of the census. The age and birth year of living, coresident children were directly observed; those for deceased and living, non-coresident children were imputed.^3^

When mothers’ reconstructed birth histories are limited to births occurring in the 5 years preceding each census, all deceased children are assumed to have died before the age of 5. Other measures of child mortality, in contrast, include ‘children’ who were adults at the time of their death. More importantly, our selection criteria allow us to reduce potential bias from unobserved migration and potential endogeneity between segregation and mortality. Details of the imputation procedure—which relies on each mother’s age, the ages of her coresident children, the age intervals between her surviving children, age-specific fertility rates of women in her birth cohort, age-specific mortality rates for children by birth cohort, age-specific rates of children leaving home, and other characteristics of the mother and her spouse to probabilistically assign birth years to deceased and non-coresident children—are provided elsewhere (Hacker, 2020; Luther & Cho, 1988). Briefly, because reconstructed birth histories rely on more information, including large age gaps between surviving children suggestive of the birth of a child who died before the census, they result in a better estimate of when deceased children were born, when they were exposed to the risk of death, and when they died. Although the imputation procedure is probabilistic and subject to uncertainties, the overall number of child deaths experienced by mothers are unaffected; the reconstruction procedure is used only to establish the likely timing of deceased children’s births. We provide a longer discussion of the advantages of the relying on reconstructed birth histories in Appendix S1. We also compare the results of our analysis using the more commonly used mortality index as the dependent variable (model results are similar) and the robustness of the results to different assumptions about differentials in census under-enumeration among Black and White children.

### 2.2 | Measurement of segregation

Research on contemporary populations suggests that pathways linking residential segregation and population health are complex and likely vary by the dimension of segregation measured (Massey & Denton, 1988) and geographic level of aggregation. There is a large methodological literature on advantages and disadvantages of various measures, best choices of measurement levels (including choice of macro and micro geographic units), how to incorporate spatial components, and the usefulness of different segregation indexes as independent variables in social analyses and health research (Acevedo-Garcia et al., 2003; Kramer et al., 2010; Massey & Denton, 1988; Reardon & O’Sullivan, 2004; Xu et al., 2014). Unfortunately, limitations in the historical IPUMS data—which identify individuals’ state, county, city, and ED of residence, but are not geocoded to their exact locations—do not allow us to construct more advanced segregation measures proposed in the literature. The location of census EDs and their boundaries, for example, are unknown for most cities and rural areas. Addresses were collected for most urban residents in 1900 and 1910 but only a few have been geo-coded.^4^ No address information was collected for rural residents.

The complete-count historical census data allow the construction of a few segregation measures at different levels of aggregation. We found that measures at the census ED level, the smallest level of geography available consistently in the census, to be the most useful for analysis of child mortality. In 1900 and 1910, census EDs typically conformed to recognisable neighbourhoods and contained about 1000–3000 individuals. EDs are available for both the rural and urban populations and are small enough to capture the segregation of groups that may appear integrated when using larger geographic units such as city wards or city-level measures of segregation such as the aspatial dissimilarity index (Cutler et al., 1999; Kellogg, 1977; Logan et al., 2015; Logan & Parman, 2017).^5^ We constructed two measures of segregation for each ED, the *SIS* (Grigoryeva & Ruef, 2015) and the proportion of households that were headed by Black individuals (*proportion Black*). The latter measure is considered a proxy measure of segregation because it is constructed without consideration for the spatial distribution of races within the ED and is independent of the racial composition and residential patterning in the larger geographic unit (Kramer & Hogue, 2009). In addition to reflecting segregation, *proportion Black* likely captures aspects of socioeconomic status and neighbourhood conditions that may have been imperfectly captured or unmeasured by other census variables such as occupation, homeownership, unemployment, and literacy.

We also used EDs to construct traditional aspatial city-level measures of racial evenness and isolation (the dissimilarity and isolation indexes, *DI* and *II*) and used the measures in mortality analyses of the South’s urban population (results shown in a separate Appendix S1). We limit our presentation here to analyses based on the ED-level variables *SIS* and *proportion Black* for several reasons. First, as shown in Figure 1, the dissimilarity and isolation indexes suggest relatively low levels of urban segregation at the turn of the century, despite known high levels of social segregation between Blacks and Whites and high levels of within-neighbourhood spatial segregation (Grigoryeva & Ruef, 2015). Aspatial city-level measures, in other words, masked significant heterogeneity within geographic subunits, and therefore have significant limitations in the historical context of this study. Second, unobserved but suspected large differences in the disease and mortality environment across southern cities likely bias intercity comparisons (see Figure 2 below for the large spatial differentials in mortality across southern counties). As discussed in more detail below, we employ county-level fixed effects to control for unobserved heterogeneity in the disease environment in our regression analyses. Unfortunately, the small number of southern cities in the early 20th-century South relative to the number of southern counties (e.g., the 1900 dataset identifies only 516 urban places in the South’s 1353 counties) precluded the use of county-level fixed effects in models using city-level measures of dissimilarity and isolation. In the Appendix S1, we show the results of regression models using *DI* and *II* and state-level fixed effects models. Although the results of these models appear reasonable—all else being equal a higher *DI or II* was typically associated with modestly higher child mortality among Black children and little difference among White children—we deemed these results subject to much greater potential bias from unobserved heterogeneity within states. In addition, unlike the ED-level variables *proportion Black* and *SIS*, the inclusion of *DI* and *II* in models of child mortality resulted in some degradation of the overall model performance. As shown in the Appendix S1, models with *DI* and *II* had larger Black–White differentials in mortality relative to the baseline models than models with *proportion Black* and *SIS* alone and did not result in improvements to model *R*^2^ values.

Grigoryeva and Ruef’s (2015) *SIS* is based on the sequential ordering of households by race in the original census enumeration. Before 1960, censuses were conducted by enumerators going door-to-door and recording the residents of each household. The order in which households were enumerated was preserved on the manuscript census returns and in the IPUMS complete-count datasets. Using geocoded data for Washington, D.C. in 1880, Grigoryeva and Ruef demonstrated that the order of households in the original manuscripts was consistent with an enumerator walking along typical travel paths. They concluded that households adjacent to each other in the manuscript returns were typically next door or at least physically very close to one another. Segregation within the ED could therefore be measured using the sequential ordering of the race of household heads in the ED:

$$SIS=1-\frac{R-2}{E(R)-2},$$

where R is the observed number of race-specific ‘runs’ of household heads in the ED and E(R) is the expected number of runs that would occur if the sequential patterning observed was due to randomness. A new run is counted for every change in the race of the household head observed from the previous household head. For example, if “B” designates a Black-headed household and “W” a White-headed household, a sequential ordering of W–W–W–W–B–B–B–B households in the manuscript returns is equivalent to two race-specific runs, while a sequence of W–B–W–B–W–B–W–B households is equivalent to eight runs. A SIS value of 1.0 indicates complete segregation and a value of 0.0 indicates random integration.^6^ In an ED with a SIS value of 1.0, a person walking the same route as the census enumerator would encounter all households of one race first, presumably living next door to one another, and then all households of the other race second, also presumably living next door to one another. In an ED with an SIS of 0.0, a person walking the same route as the enumerator would encounter different race households randomly according to their percentage in the ED. The measure can be applied to small areas with small minority populations with high precision, provided there are five or more minority households in an area. The SIS allows to us to capture what Grigoryeva and Ruef term ‘tertiary segregation’, when social groups remain socially segregated despite living in close spatial proximity to one another.

## 3 | ANALYTICAL DESIGN AND RESULTS

We begin by constructing complete birth histories of currently married women in the 1900 and 1910 IPUMS complete-count datasets according to the methods detailed by Luther and Cho (1988) and Hacker (2020). Our restriction to children born to married couples allowed us to include father’s occupation, employment, weeks unemployed, homeownership, and literacy in our descriptive analysis and as covariates in our models. Because we lacked race information for deceased children, we imputed children’s race from their mother’s and father’s race. When constructing empirical models, we further limited the analytical dataset to White and Black children born in the 5 years before each census, whose parents reported marital durations of 5 or more years, lived in southern census regions, and lived in census EDs with 5 or more households headed by individuals of each race. The latter restriction ensures that *SIS* can be measured without significant error (Grigoryeva & Ruef, 2015).

We used logistic regression to model the relationship between child mortality and racial residential segregation. Because urban and rural areas in the early 20th century were characterised by large differences in economies, occupations, disease environments, and child mortality levels, we constructed separate models for the rural and urban populations and combined rural–urban models for each census year. The dependent variable measures whether a child born in the 5 years before the census was alive or deceased on the day of the enumeration (coded as 0 and 1, respectively).^7^ Independent variables of interest in our analysis included a dummy variable for children’s race Black (coded as 0 for White children and 1 for Black children), the proportion of households in each ED headed by Black men and women (*proportion Black*), the *SIS*, and variables interacting children’s race with *proportion Black* and *SIS* to determine whether the relationship between segregation and child mortality varied by the race of the child. We centred *proportion Black* and *SIS* to their mean values in each model and used the centred values in the construction of the interaction variables, which allows the interaction effects to be interpreted at their mean values. Other independent variables in the models included each child’s birth order and number of years born before the census; mother’s age at birth and labour force participation; father’s occupation group, homeownership, unemployment (1910 only), and weeks unemployed (1910 only); and parents’ literacy (coded 1 if both parents could read and write and 0 if only one parent or neither parent could read and write).

Our analytical strategy was to construct baseline models of child mortality without the segregation variables—replicating prior scholarship as closely as possible—to observe race differentials in mortality (Preston et al., 1994; Preston & Haines, 1991). We then introduced our segregation variables separately and in combination to examine: (1) the association between each segregation variable and the mortality of Black and White children; and (2) how race differences in child mortality observed in the baseline models were affected by the addition of the segregation variables.

The large number of cases and EDs in the IPUMS complete-count datasets allowed us employ area fixed effects models to control from unobserved heterogeneity across geographic areas. As noted by other researchers (e.g., Dribe et al., 2020; Elman et al., 2019), geographic differentials in mortality in the early 20th century were large and could vary dramatically between adjacent areas, likely reflecting differences in local disease environments. The prevalence of malaria and tuberculosis, for example, varied significantly across regions and states (Carney, 1874). Figure 2 maps our estimates of White and Black child mortality in southern counties with 30 or more births in the 5 years before the 1910 census. Mortality rates were significantly higher for Black children than for White children in each county. Within each race, child mortality rates were noticeably higher in counties located on or near the lower Mississippi River and its tributaries, where flooding and ponding provided ideal breeding areas for the *Anopheles* mosquito and the transmission of malaria (see correspondence with Elman et al., 2019, Figure 2, and Carney, 1874). Every state, however, had areas of high and low mortality. Empirical models that do not control for the heterogeneity of local disease environments are at greater risk of conflating the relationship between racial residential segregation and other explanatory variables that varied across geographic areas with the effect of the disease environment. By employing county-level fixed effects, we held geographic variations in mortality fixed and estimated the relationship between racial residential segregation and child mortality within counties. In all models we clustered standard errors at the ED level.

### 3.1 | Descriptive results

Table 1 presents the mean proportion of children dying in the 5 years before the 1900 and 1910 censuses by race in various categories (the percentage of White and Black children in each category is shown in parentheses) using the same selection criteria used in the models. Overall, 24.4% of Black children born in the five years before the 1900 census whose mothers resided in rural areas died before the census was conducted, compared with 15.5% of the White children. In urban areas, the percentages dying were much higher: 36.2% of Black children and 20.1% of White children died before the 1900 census. In 1910 the patterns were similar, but with significantly lower mortality in urban areas, especially for White children. Although Black children experienced a much greater risk of death than White children in both urban and rural areas, the differential was greater in urban areas. In rural areas, for example, Black children experienced a 58% higher risk of death than White children in 1900 and a 60% higher risk of death in 1910; in urban areas, they experienced an 80% higher risk of death in 1900 and a 94% higher risk in 1910.

Both Black and White children born to literate parents experienced a lower risk of death compared to children born to illiterate parents of the same race in both urban and rural areas. But Black children were more likely to have one or more illiterate parents than White children. In 1900, for example, 71.5% of rural Black children in the South had one or more illiterate parents compared to just 25.7% of rural White children in the South. In both census years and in both rural and urban areas, Black children were more likely than White children to have mothers in the paid labour force, fathers with lower status and lower paid occupations such as general labourers, and parents who rented rather than owned their own homes. These structural inequities were associated with higher child mortality rates, although we cannot be confident about the existence of causal relationships or the direction of the relationships.

Tables 2 and 3 summarise child mortality by race in the 1900 and 1910 censuses for the 33 southern cities with 20,000 or more residents in 1900 and with 5% or more Black residents. We also show the mean *SIS* and the proportion of the population that was Black. Among Black children, the highest mortality in both censuses was observed in Savannah, Georgia where a staggering 47.4% of Black children born in the 5 years before the 1900 census died before the enumeration (in 1910, the corresponding percentage was 48.2). Other cities with high Black mortality rates included Charleston, South Carolina, Macon, Georgia, and Little Rock, Arkansas. The lowest level of Black child mortality among the 33 cities was in Austin, Texas (where 25.2% had died before the 1900 census and 22.3 before the 1910 census). In all cities, Black child mortality was dramatically higher than White child mortality, in most cases more than twice the White level. The largest mortality differential was observed in Savannah, Georgia in 1910—where Black children were 3.2 times more likely to die before the census than White children—while the lowest mortality differentials were observed in Austin and San Antonio.

### 3.2 | Regression results

To examine the potential relationship between racial residential segregation and child mortality, we constructed logistic regression models controlling for known correlates of mortality. In Table 4 we show selected regression results for children born in the 5 years before the 1900 census. Models 1–4 are limited to the rural populations, Models 5–8 are limited to the urban populations, and Models 9–12 are for rural and urban areas combined. The first model for each area—Models 1, 5, and 9—show baseline regression results without segregation variables. Models 2–4, 6–8, and 10–12 add the ED-level segregation variables *proportion Black* and *SIS* separately and in combination. In Table 5 we show results of similar logistic regression models for children born in the 5 years before the 1910 census.

For ease of interpretation, we show exponentiated coefficients (odds ratios), which indicate the increase in the odds of children’s death relative to the reference category. For example, the results for Model 1 in Table 4—our baseline model of the rural population in 1900—indicate that Black children born in the 5 years before the 1900 census were 1.263 times more likely to have died before the enumeration than White children, even after controlling for literacy, occupation, homeownership, and unemployment. Model 5 indicates that Black children in urban areas were 1.656 times more likely to have died, all else being equal. The corresponding values in Table 5 for the 1910 census was 1.325 in rural areas and 1.779 in urban areas. Quite clearly, Black children in southern cities experienced a significantly higher relative risk of death than White children.

Coefficients for other variables in the model are consistent with prior studies (Dribe et al., 2020; Preston & Haines, 1991; Preston et al., 1994) and are not shown. Parental literacy and fathers with higher status and pay occupations were associated with greater survival for both White and Black children in all models. Children of unemployed fathers experienced higher mortality rates relative to children of employed fathers, while children whose parents owned their home experienced lower mortality than children of parents who rented. In the urban models, children of parents whose residence was in a larger urban area (populations of 25,000 or more) had no significant difference in the risk of mortality relative to children of parents whose residence was in a smaller urban area, but in combined models the coefficients indicate significantly higher child mortality in urban areas relative to rural areas.

Models 2–4, 6–8, and 10–12 add the ED-level segregation measures singly and in combination. The results indicate that racial residential segregation was positively correlated with child mortality in both rural and urban areas but with some differences by measure, type of area, children’s race, and census year. The proportion of households headed by Black individuals was positively and strongly correlated with the mortality of Black children in all models for both rural and urban areas and in both census years. It was also positively—although more modestly—correlated with the mortality of White children in rural areas only. Although our cross-sectional data do not allow us to determine causal relationships or distinguish among possible pathways connecting the racial composition of EDs with child mortality, White and Black individuals living in EDs with higher proportions of Black-headed households likely experienced one or more of the health challenges typically associated with residents of minority neighbourhoods today—lower quality and crowded housing that facilitated the spread of infectious disease, poorer environmental conditions, more crime, and limited access to high paying jobs—relative to residents in EDs with lower proportions of Black-headed households.

As noted earlier, *proportion Black* does not depend on the spatial patterning of households within EDs and is therefore considered a proxy measure of racial residential segregation. In addition to reflecting segregation, the proportion of Black households in an ED could be capturing aspects of individuals’ socioeconomic status and economic wellbeing imperfectly captured by other variables in the model. In other words, omitted variables (e.g., income, wealth, or some other variable) could be acting as confounders, biasing the estimated relationship between *proportion Black* and child mortality. Hypothetically, if we were able to include better measures of parents’ economic wellbeing in the models, the results might indicate that the positive relationship between *proportion Black* and child mortality shown in Models 2–4, 6–8, and 10–12 was spurious. Although we cannot dismiss that possibility, a spurious relationship is unlikely for several reasons. First, our regression models include controls for multiple measures of socioeconomic status. Occupation, in particular, has been shown to be strongly correlated with income, wealth, and child mortality (Dribe et al., 2020; Hacker et al., 2023; Sobek, 1995). Together with homeownership, unemployment, and literacy, occupation likely controls for variations in economic wellbeing across EDs. Second, our use of county-level fixed effects, which controls for unobserved heterogeneity across counties, also reduces the risk of omitted variable bias. Finally, *proportion Black* and child mortality were very strongly correlated. The strength of the association suggests that omitted variable bias would need to be very large to render the observed relationship spurious.

In contrast to *proportion Black*, *SIS* depends on the spatial patterning of households with EDs, measuring segregation from the sequential race patterning of household heads. Correlations between *SIS* and child mortality may therefore reflect different pathways connecting residential segregation and mortality. The results indicate that *SIS* segregation was not correlated with the mortality of White children in rural or urban areas in both censuses. When both rural and urban areas are combined, however, *SIS* was negatively correlated with the mortality of White children in several of the models, suggesting a possible benefit of segregation for the White population.

Importantly, in most models—those for rural places in both the 1900 and 1910 censuses and those for urban places in the 1910 census—the odds ratio for the interaction of *SIS* and *child’s race Black* was well above 1.0 and statistically significant, indicating that within-ED segregation was positively correlated with the risk of death of Black children. The coefficients for *proportion Black* and *SIS* and their interactions with children’s race were similar in models when each was included as the sole segregation variable and in models with both segregation variables included. Overall, these findings are consistent with research on modern populations, where racial residential segregation has been associated with negative effects on Black population health and no or even possible beneficial effects on White population health (Beaulieu & Continelli, 2011; Massey, 1996; McFarland & Smith, 2011).

An additional indication of the importance of racial residential segregation to child mortality is the reduction in the size of the coefficients associated with Black children relative to the reference group of White children after the addition of segregation variables to the model. Earlier researchers hypothesised that the large unexplained race differentials in child mortality were the result of income differentials not controlled for by occupation and other independent variables in the models (e.g., Preston et al., 1994). Although we do not dispute this possibility, our results show that racial residential segregation was responsible for a significant proportion of the race disparities in child mortality unexplained in earlier studies. For southern Black children in 1900, the coefficient was reduced from 1.263 to 1.205 in rural areas and from 1.656 to 1.593 in urban areas. These changes represent a reduction in the unexplained differential between the mortality of Black and White children in the baseline models of 22.1% in rural areas and 9.6% in urban areas. In 1910, racial residential segregation explained 15.7% of the race differences in the baseline model for rural areas and 13.0% in urban areas. If we consider literacy, occupation, home-ownership, unemployment, and other variables in the model to be products of segregation rather than independent variables, the contribution of segregation to the large race differentials in child Characteristics of child mortality observed in the early 20th century would be higher still. Importantly, however, most of the reduction in unexplained race differentials comes from the addition of *proportion Black* to the baseline models. The addition of *SIS* to the models played a relatively minor role in reducing the unexplained race differentials in 1910 and an even smaller role in 1900.

Our results suggest that residential segregation was more important to race disparities in child mortality in rural areas than in urban areas, highlighting a need for further research into segregation in rural areas, where more than 80% of the southern population lived at the turn of the 20th century. Although there has been remarkably little attention to rural segregation per se (see, however, Lichter et al., 2007; Logan & Parman, 2017), economists, historians, and geographers have long stressed that the rural Black population’s severe poverty resulted in high rates of tenancy and sharecropping and isolation to areas with poor soil quality, drainage, low agricultural productivity, and endemic malaria (Du Bois, 1901; Elman et al., 2019; Fisher, 1973; Ransom & Sutch, 1977).

Finally, Table 6 shows the results for models combining rural and urban areas and pooling observations from both censuses with a dummy variable for census year. The results, which are based on the mortality or survival of approximately 4.7 million children, indicates the *proportion Black* was modestly correlated with the increased risk of death among White children while *SIS* was negatively correlated with White children’s mortality. Both variables were strongly and positively correlated with the risk of death among Black children. Adding both segregation variables to the pooled model reduced the observed race difference in mortality from a 36.9% higher risk of death among Black children in the baseline model to a 29.8% higher risk of death, representing a decline in the race differential unexplained by the variables in the baseline model of 19.2%.

Overall, the results indicate that increased racial residential segregation was consistently and strongly associated with the higher morality of Black children at the turn of the 20th century. Rather than being associated with better outcomes in the Black population (e.g., Collins & Margo, 2000; Logan & Parman, 2018), we find that racial residential segregation was associated with lower survival probabilities among Black children, consistent with theories that residential segregation, and structural racism more generally, was a fundamental cause of long-standing racial inequities in population health.

## 4 | CONCLUSION

Public health researchers in the United States consider racial residential segregation—the degree to which Black and White individuals in a geographic area live separately from on another—to be a spatial manifestation of structural racism and a ‘fundamental’ cause of persistent racial disparities in health and mortality, with its impacts being particularly salient for children (Phelan & Link, 2015; White & Borrell, 2011; Williams & Collins, 2001). Although historical policies contributing to racial residential segregation are now illegal, trends and patterns in segregation beginning in the late 19th century continue to impact population health, particularly the health and longevity of Black individuals. The clustering of poverty and socioeconomic disadvantage within Black communities (and the clustering of socioeconomic advantage within White communities) has been shown to have significant ‘neighbourhood effects’ on residents’ health and mortality, independent of their individual-level characteristics (Acevedo-Garcia & Osypuk, 2008; Almond et al., 2006; Cutler & Glaeser, 1997; Cutler et al., 1999; Massey & Denton, 1993; Williams & Collins, 2001). Although the causal pathways linking racial residential segregation to health and mortality are complex and difficult to isolate, they are likely multiple, overlapping, and vary across temporal and geographic contexts (Kramer & Hogue, 2009; McFarland & Smith, 2011).

In a recent review of published studies, Phelan and Link (2015) contended that if racism is a fundamental cause of race inequalities of health and mortality, ‘these inequalities should endure despite changes over time in diseases, risk, and protective factors and medical interventions, because mechanisms that have been reduced or eliminated are replaced by new mechanisms’. Their findings suggest that systemic racism influences a variety of flexible resources, resulting in the persistence of white advantages in health and mortality under a wide range of circumstances and through multiple replaceable pathways. Likewise, if racial residential segregation is a fundamental cause of race disparities in health and mortality (Williams & Collins, 2001), we would expect a similar relationship between residential segregation and population health in different contexts across time and space.

This paper examined the relationship between racial residential segregation and child mortality in a dramatically different context than most contemporary studies: the U.S. South at the turn of the 20th century. During this period child mortality rates were very high—more than 20 times the average rate experienced in the U.S. today—and infectious diseases were the primary killers of children and adults. A few researchers (e.g., Troesken, 2004) have hypothesised that relatively low levels of racial segregation in urban areas in the early 20th century—as measured by conventional measures of racial evenness and isolation—made some types of place-based discrimination more difficult to practice. Public investments in water supply and sewage systems, for example, which were increasingly made by U.S. cities in the late 19th and early 20th centuries, benefitted Blacks and Whites alike (Troesken, 2002). Other research has suggested the segregation before the late 20th may have had beneficial impacts on the Black population (e.g., Collins & Margo, 2000; Logan & Parman, 2018), casting some doubt about the possible role of racial residential segregation as a long-standing fundamental cause of racial disparities in health and mortality.

Using newly constructed 1900 and 1910 IPUMS complete-count datasets (Ruggles et al., 2021), we evaluated the relationship between racial residential segregation and the mortality of 4.7 million Black and White children born to mothers residing in southern census regions. Consistent with earlier scholarship (Preston et al., 1994), our baseline regression models revealed large race disparities in child mortality that varied between rural and urban areas, even after controlling for a wide range of demographic and socioeconomic variables. We evaluated the association between child mortality and two measures of racial residential segregation: the proportion of households in each census ED headed by Blacks (*proportion Black*) and the *SIS* for each district. Regression models included multiple control variables for parents’ socioeconomic status (occupation, homeownership, unemployment, and literacy) and relied on county-level fixed effects, increasing confidence that results were not significantly biased by omitted variables (e.g., income and wealth). Both segregation variables were positively and strongly associated with the mortality of Black children in most models. The relationship between *proportion Black* and the mortality of White children was also positive but more modest. We found no relationship between *SIS* and mortality of White children in most models and a negative relationship in a few models. The addition of the two segregation variables to the baseline models reduced unexplained race differentials in child mortality by between 9.6% and 22.1%, depending on the census year and type of area, suggesting that racial residential segregation was an important factor in children’s risk of death in the early 20th century, particularly the risk of death experienced by Black children.

Although these findings are limited to the turn of the 20th-century South, the results suggests that racial residential segregation had a similar relationship with disparities in mortality in the past as it does with race disparities in population health and mortality today. Given the dramatically different context of the South more thana century ago—much lower levels of urbanisation, lower macro-level (city) but higher micro-level (ED) levels of racial residential segregation, limited or lacking water supply and sewage systems, and higher proportions of deaths from infectious diseases—it is likely that the pathways connecting segregation with child mortality in the past differed from the pathways connecting segregation and population health today. Although we cannot identify causal pathways and how they may have changed across time and space, the similar overall association between racial residential segregation and child mortality in these dramatically different contexts supports the hypothesis that racial residential segregation, and systemic racism more generally, were long-standing fundamental causes of inequities in health and mortality (Feagin & Bennefield, 2014; Phelan & Link, 2015).

## Supplementary Material

appendix

## Figures and Tables

**FI GURE 2:**
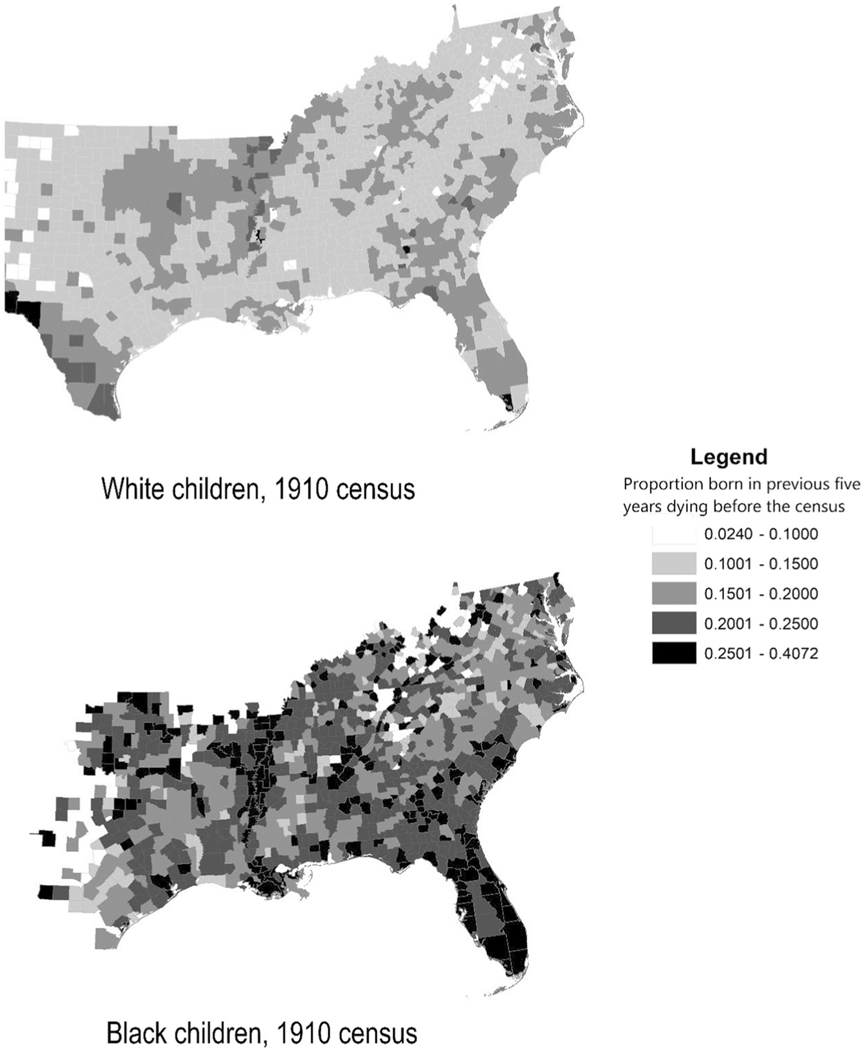
Mortality of White and Black children, 1905–1910.

**TABLE 1:** Proportion of children born 5 years prior the 1900 and 1910 censuses dying before the census.

	Rural areas				Urban areas			
Census year	1900		1910		1900		1910	
Race of child	White	Black	White	Black	White	Black	White	Black
Parent’s Literacy								
One or both Illiterate	0.176 (25.7)	0.258 (71.5)	0.186 (19.3)	0.273 (53.0)	0.245 (9.7)	0.372 (49.9)	0.236 (7.7)	0.407 (30.8)
Both literate	0.147 (74.3)	0.209 (28.5)	0.147 (80.7)	0.218 (47.0)	0.194 (90.3)	0.306 (50.1)	0.172 (92.3)	0.316 (69.2)
Mother’s labour force								
Not in paid labour force	0.154 (97.1)	0.238 (81.3)	0.154 (88.3)	0.236 (49.4)	0.197 (97.3)	0.322 (70.4)	0.174 (94.6)	0.307 (55.8)
In paid labour force	0.174 (2.9)	0.269 (18.7)	0.162 (11.7)	0.258 (50.6)	0.278 (2.7)	0.380 (29.6)	0.237 (5.4)	0.391 (44.2)
Father’s occupation								
Professional, technical	0.148 (1.3)	0.248 (0.6)	0.137 (0.9)	0.242 (0.5)	0.169 (5.0)	0.293 (3.1)	0.142 (4.9)	0.302 (3.0)
Farmer	0.148 (70.0)	0.237 (65.4)	0.151 (69.0)	0.242 (68.2)	0.201 (2.3)	0.303 (2.3)	0.196 (1.6)	0.334 (1.5)
Managers, officials, Prop.	0.163 (2.4)	0.291 (0.2)	0.153 (2.7)	0.276 (0.2)	0.183 (13.5)	0.316 (1.7)	0.168 (17.2)	0.325 (2.5)
Clerical and sales	0.149 (0.9)	0.290 (0.1)	0.140 (1.6)	0.278 (0.1)	0.176 (9.8)	0.315 (1.2)	0.152 (14.5)	0.314 (2.1)
Craftsmen	0.172 (4.4)	0.291 (1.2)	0.164 (5.4)	0.275 (1.2)	0.202 (24.6)	0.344 (10.6)	0.181 (27.8)	0.344 (12.3)
Apprentices, operatives	0.188 (3.6)	0.276 (2.2)	0.176 (4.9)	0.286 (2.2)	0.209 (11.6)	0.343 (13.5)	0.183 (13.6)	0.353 (13.2)
Service workers	0.185 (0.3)	0.281 (0.4)	0.185 (0.4)	0.276 (0.5)	0.213 (3.7)	0.315 (9.4)	0.196 (4.1)	0.319 (11.9)
Farm labourers	0.161 (5.6)	0.251 (14.6)	0.156 (6.5)	0.249 (14.5)	0.222 (0.5)	0.329 (2.8)	0.197 (0.7)	0.354 (3.4)
General labourers	0.178 (7.5)	0.262 (11.6)	0.170 (7.9)	0.262 (12.0)	0.211 (24.9)	0.349 (51.8)	0.201 (14.3)	0.352 (49.4)
Nonoccupational response	0.158 (4.1)	0.232 (3.7)	0.171 (0.7)	0.265 (0.5)	0.203 (4.1)	0.319 (3.8)	0.219 (1.3)	0.381 (0.9)
Unemployment								
Employed			0.154 (97.0)	0.247 (97.7)			0.176 (96.8)	0.343 (96.0)
Unemployed			0.162 (3.0)	0.261 (2.3)			0.213 (3.2)	0.381 (4.0)
Homeownership								
Rents	0.161 (48.9)	0.245 (77.0)	0.159 (53.8)	0.248 (78.0)	0.203 (66.7)	0.346 (77.2)	0.181 (66.2)	0.347 (75.5)
Owns or mortgage	0.148 (51.1)	0.240 (23.0)	0.150 (46.2)	0.246 (22.0)	0.191 (33.3)	0.318 (22.8)	0.170 (33.8)	0.335 (24.5)
City size (population)								
2500–9999					0.170 (33.4)	0.323 (32.9)	0.170 (33.4)	0.323 (32.9)
10,000–24,999					0.173 (15.4)	0.328 (17.2)	0.173 (15.4)	0.328 (17.2)
25,000–99,999					0.183 (22.5)	0.377 (23.2)	0.183 (22.5)	0.377 (23.2)
100,000+					0.183 (28.7)	0.352 (26.8)	0.183 (28.7)	0.352 (26.8)
Mean proportion dying	0.155	0.244	0.155	0.247	0.201	0.362	0.177	0.344
Number of cases	1,152,580	712,994	1,253,904	744,847	241,566	105,618	332,048	133,895

*Note:* The analytical dataset includes White and Black children born in the 5 years before each census, whose mothers were: (i) enumerated with valid children ever born and children surviving data; (ii) enumerated in a residence location that could be clearly identified as urban or rural; (iii) currently married at the time of the census; (iv) living in a southern census region; (v) living in a census enumeration district with five or more Black and five or more White-headed households; (vi) in marriages having a duration of five or more years; and (vii) living in the same household as their husbands. See Hacker (2020) for details on the reconstruction of women’s complete birth histories. The proportional values shown indicate the proportion of children born in the 5 years before each census who died before the date of the census, while the values in parentheses indicate the percentage of children in each category. For example, 25.7% of White children born in the 5 years before the 1900 census were born to one or two illiterate parents, while 74.3% were born to two literate parents. Among the former group, the proportion who died before the census was 0.176 (or 17.6%). Among the latter group of children, 14.7% died.

*Source:* 1900 and 1910 Complete Count IPUMS datasets (Ruggles et al., 2021).

**TABLE 2:** Descriptive statistics for southern cities in 1900 with 20,000 or more total residents and 5% or more black residents.

	Proportion dying		Number of enum. districts (EDs)	Seq. index of segregation (SIS)	Total population (1900)	Proportion Black
	White	Black				
Baltimore, Maryland	0.196	0.350	319	0.471	508,957	0.156
New Orleans, Louisana	0.199	0.357	149	0.456	287,104	0.271
Washington, DC	0.172	0.326	148	0.653	278,718	0.311
Louisville, Kentucky	0.161	0.355	144	0.564	204,731	0.191
Memphis, Tennessee	0.174	0.329	62	0.641	102,320	0.488
Atlanta, Georgia	0.200	0.352	47	0.682	89,872	0.398
Richmond, Virginia	0.180	0.320	55	0.540	85,050	0.379
Nashville, Tennessee	0.183	0.376	48	0.622	80,865	0.372
Wilmington, Delaware	0.198	0.375	46	0.476	76,508	0.127
Charleston, South Carolina	0.197	0.433	41	0.509	55,807	0.565
Savannah, Georgia	0.213	0.474	47	0.526	54,244	0.518
San Antonio, Texas	0.186	0.254	35	0.347	53,321	0.141
Norfolk, Virginia	0.196	0.358	34	0.512	46,624	0.434
Houston, Texas	0.172	0.293	29	0.492	44,633	0.327
Covington, Kentucky	0.170	0.424	39	0.492	42,938	0.058
Dallas, Texas	0.185	0.341	38	0.486	42,638	0.212
Augusta, Gerogia	0.250	0.393	22	0.648	39,441	0.469
Mobile, Alabama	0.191	0.366	19	0.469	38,469	0.443
Birmingham, Alabama	0.168	0.387	28	0.645	38,415	0.431
Little Rock, Arkansas	0.195	0.410	25	0.622	38,307	0.384
Galveston, Texas	0.170	0.320	30	0.359	37,789	0.219
Knoxville, Tennessee	0.192	0.270	16	0.462	32,637	0.225
Montgomery, Alabama	0.166	0.363	16	0.608	30,346	0.570
Chattanooga, Tennessee	0.202	0.379	18	0.671	30,154	0.435
Jacksonville, Florida	0.176	0.382	14	0.588	28,429	0.571
Fort Worth, Texas	0.162	0.330	26	0.435	26,688	0.159
Lexington, Kentucky	0.178	0.319	14	0.650	26,369	0.384
Macon, Georgia	0.218	0.396	16	0.699	23,272	0.496
Austin, Texas	0.156	0.252	20	0.467	22,258	0.262
Roanoke, Virginia	0.140	0.276	13	0.597	21,495	0.271
Columbia, South Carolina	0.213	0.369	12	0.556	21,108	0.467
Wilmington, North Carolina	0.191	0.342	15	0.543	20,976	0.496
Waco, Texas	0.188	0.354	11	0.587	20,686	0.282

*Note*: Proportion dying is the proportion of White and Black children born in the 5 years before the census who died before the nominal date of the census (1 June 1900). The Sequence Index of Segregation was estimated according to methods outlined by Grigoryeva and Ruef (2015) and described in the text. Proportion Black is the proportion of each city’s population in the 1900 census enumerated as Black.

*Source*: Ruggles et al. (2021); Gibson and Jun (2005).

**TABLE 3:** Descriptive statistics for southern cities in 1910 with 20,000 or more total residents in 1900 and 5% or more Black residents.

	Proportion dying		Number of enum. districts (EDs)	Seq. index of segregation (SIS)	Total population (1900)	Proportion Black
	White	Black				
Baltimore, Maryland	0.176	0.322	397	0.429	558,485	0.152
New Orleans, Louisana	0.177	0.333	245	0.474	339,075	0.263
Washington, DC	0.136	0.283	241	0.630	331,069	0.285
Louisville, Kentucky	0.154	0.353	194	0.559	223,928	0.181
Atlanta, Georgia	0.169	0.328	84	0.749	154,839	0.335
Birmingham, Alabama	0.155	0.387	63	0.869	132,685	0.394
Memphis, Tennessee	0.170	0.401	198	0.754	131,105	0.400
Richmond, Virginia	0.157	0.298	93	0.657	127,628	0.366
Nashville, Tennessee	0.161	0.355	73	0.719	110,364	0.331
San Antonio, Texas	0.186	0.261	63	0.379	96,614	0.111
Dallas, Texas	0.147	0.331	60	0.472	92,104	0.196
Wilmington, Delaware	0.169	0.357	50	0.510	87,411	0.104
Houston, Texas	0.142	0.338	63	0.588	78,800	0.304
Fort Worth, Texas	0.172	0.291	62	0.399	73,312	0.181
Norfolk, Virginia	0.158	0.380	50	0.661	67,452	0.371
Savannah, Georgia	0.151	0.482	45	0.794	65,064	0.511
Charleston, South Carolina	0.174	0.396	48	0.748	58,833	0.528
Jacksonville, Florida	0.166	0.386	26	0.809	57,699	0.508
Covington, Kentucky	0.137	0.286	45	0.394	53,270	0.054
Mobile, Alabama	0.148	0.363	40	0.555	51,521	0.442
Little Rock, Arkansas	0.155	0.403	29	0.733	45,941	0.316
Chattanooga, Tennessee	0.160	0.366	33	0.789	44,604	0.402
Augusta, Gerogia	0.213	0.358	24	0.887	41,040	0.447
Macon, Georgia	0.202	0.391	27	0.910	40,665	0.446
Montgomery, Alabama	0.144	0.373	19	0.769	38,136	0.507
Galveston, Texas	0.158	0.329	31	0.386	36,981	0.217
Knoxville, Tennessee	0.178	0.270	24	0.523	36,346	0.210
Lexington, Kentucky	0.127	0.290	24	0.707	35,099	0.314
Roanoke, Virginia	0.127	0.327	25	0.590	34,874	0.227
Austin, Texas	0.141	0.223	20	0.585	29,860	0.250
Waco, Texas	0.148	0.297	15	0.763	26,425	0.230
Columbia, South Carolina	0.157	0.380	14	0.938	26,319	0.439
Wilmington, North Carolina	0.138	0.349	16	0.908	25,748	0.470

*Note*: Proportion dying is the proportion of White and Black children born in the 5 years before the census who died before the nominal date of the census (15 April 1910). The Sequence Index of Segregation was estimated according to methods outlined by Grigoryeva and Ruef (2015) and described in the text. Proportion Black is the proportion of each city’s population in the 1910 census enumerated as Black or Mixed Race.

*Source*: Ruggles et al. (2021); Gibson and Jun (2005).

**TABLE 4:** Logistic regression models of child mortality in reconstructed birth histories, southern census regions, 1900 census.

	Rural areas				Urban areas				Combined rural and urban areas			
Model number	(1)	(2)	(3)	(4)	(5)	(6)	(7)	(8)	(9)	(10)	(11)	(12)
Child’s race												
White	*ref.*	*ref.*	*ref.*	*ref.*	*ref.*	*ref.*	*ref.*	*ref.*	*ref.*	*ref.*	*ref.*	*ref.*
Black	1.263*”	*1.212* ***	1.261***	1.205***	1.656***	1.592***	1.656***	1.593***	1.330***	1.274***	1.322***	1.258***
Residence characteristics												
Rural area									*ref.*	*ref.*	*ref.*	*ref.*
Urban city, population <25,000	-	-	-	-	*ref.*	*ref.*	*ref.*	*ref.*	1.332***	1.335***	1.303***	1.296***
Urban city, population ≥25,000	-	-	-	-	1.138	1.130	1.138	1.130	1.299***	1.31***	1.28***	1.285***
Segregation measures												
Proportion Black in ED		1.218***		1.216***		1.055		1.052		1.197***		1.192***
Child’s race Black × Prop. Black		1.313***		1.360***		1.742***		1.746***		1.285***		1.352***
Seq. index of segreg. (*S/S*)			1.022	1.034			1.006	1.019			0.949*	0.962
Child’s race Black × *SIS*			1.139***	1.231***			0.938	0.991			1.381***	1.473***
Observations	1,865,574	1,865,574	1,865,574	1,865,574	346,950	346,950	346,950	346,950	2,212,524	2,212,524	2,212,524	2,212,524
Number of enumeration districts	9471	9471	9471	9471	2240	2240	2240	2240	11,670	11,670	11,670	11,670
Number of counties	1108	1108	1108	1108	325	325	325	325	1135	1135	1135	1135
Pseudo *R*^2^	0.042	0.042	0.042	0.042	0.064	0.064	0.064	0.064	0.047	0.047	0.047	0.047
Change in race differential		19.4%	0.8%	22.1%		9.8%	0.0%	9.6%		17.0%	2.4%	21.8%

*Note*: See Table 1 for selection criteria. All models employ county-level fixed effects. Interaction variables are based on the centred mean value of *SIS* or *proportion Black* in the model. Other variables in the model include children’s year of birth and birth order; mother’s labour force participation and age at child’s birth; father’s occupation group; and couple’s literacy and homeownership. Reference categories are White children born to mothers who were not in the paid labour force; whose fathers were employed as general labourers; whose parents rented their homes, and whose parents included one or more partners who could not read and write. Change in race differential is the percentage reduction in the Black–White child mortality differential coefficient relative to baseline Models 1, 5, and 9. See Appendix S1 for full model results.

*** *p* < 0.001

** *p* < 0.01

* *p* < 0.05.

**TABLE 5:** Logistic regression models of child mortality in reconstructed birth histories, southern census regions, 1910 census.

	Rural areas				Urban areas				Combined rural and urban areas			
Model number	(1)	(2)	(3)	(4)	(5)	(6)	(7)	(8)	(9)	(10)	(11)	(12)
Child’s race												
White	*ref.*	*ref.*	*ref.*	*ref.*	*ref.*	*ref.*	*ref.*	*ref.*	*ref.*	*ref.*	*ref.*	*ref.*
Black	1.325***	1.277***	1.320***	1.274***	1.779***	1.697***	1.733***	1.678***	1.405***	1.340***	1.390***	1.333***
Residence characteristics												
Rural area	-	-	-	-	*ref.*	*ref.*	*ref.*	*ref.*	*ref.*	*ref.*	*ref.*	*ref.*
Urban city, population <25,000	-	-	-	-	1.069	1.066	1.063	1.063	1.259***	1.258***	1.229***	1.233***
Urban city, population ≥25,000									1.269***	1.271***	1.238***	1.244***
Segregation measures												
Proportion Black in ED		1.111***		1.107***		1.040		1.027		1.117***		1.122***
Child’s race Black × Prop. Black		1.377***		1.363***		1.731***		1.671***		1.377***		1.314***
Seq. index of segreg. (*S/S*)			1.019	1.024			0.999	0.990			0.957**	0.955**
Child’s race Black × *S/S*			1.159***	1.128***			1.393***	1.240***			1.401***	1.354***
Observations	1,998,751	1,998,751	1,998,751	1,998,751	465,940	465,940	465,940	465,940	2,464,691	2,464,691	2,464,691	2,464,691
Number of enumeration districts	11,441	11,441	11,441	11,441	3601	3601	3601	3601	14,960	14,960	14,960	14,960
Number of counties	1,179	1,179	1,179	1,179	454	454	454	454	1211	1211	1211	1211
Pseudo *R*^2^	0.045	0.045	0.045	0.045	0.073	0.074	0.073	0.074	0.050	0.050	0.050	0.050
Change in race differential		14.8%	1.5%	15.7%		10.5%	5.9%	13.0%		16.0%	3.7%	17.8%

*Note*: See Table 4. Additional variables in the 1910 model include father’s unemployment and weeks unemployed. See Appendix S1 for full model results.

*** *p* < 0.001

** *p*< 0.01

* *p* < 0.05.

**TABLE 6:** Logistic regression models of child mortality in reconstructed birth histories, southern census regions, pooled models.

	Rural and urban areas, 1900 and 1910 census			
Model number	(1)	(2)	(3)	(4)
Characteristics of child				
White	*ref.*	*ref.*	*ref.*	*ref.*
Black	1.369***	1.307***	1.358***	1.298***
Residence characteristics				
Rural	*ref.*	*ref.*	*ref.*	*ref.*
Urban city, population <25,000	1.290***	1.290***	1.266***	1.267***
Urban city, population ≥25,000	1.265***	1.270***	1.244***	1.250***
Segregation Measures				
Proportion Black in ED		1.162***		1.163***
Child’s race Black × Prop. Black		1.324***		1.312***
Seq. index of segreg. (*SIS*)			0.942***	0.941***
Child’s race Black × *SIS*			1.374***	1.365***
Census year				
1900	*ref.*	*ref.*	*ref.*	*ref.*
1910	1.001	1.003	0.989**	0.991*
Observations	4,677,215	4,677,215	4,677,215	4,677,215
Number of enumeration districts	26,630	26,630	26,630	26,630
Number of counties	1,232	1,232	1,232	1,232
Pseudo *R*^2^	0.047	0.047	0.047	0.048
Change in race differential		16.8%	3.0%	19.2%

*Note*: See Tables 4 and 5. See online Appendix S1 for full model results.

*** *p* < 0.001

** *p* < 0.01

* *p* < 0.05.

## Footnotes

1 States in southern census regions include Alabama, Arkansas, Delaware, District of Columbia, Florida, Georgia, Kentucky, Louisiana, Maryland, Mississippi, North Carolina, Oklahoma, South Carolina, Tennessee, Texas, Virginia, and West Virginia.

2 See Ruggles (1992) for a discussion of how unobserved migration biases age at death estimates downwards.

3 We follow Luther and Cho (1988) in referring to the ‘age’ of deceased children as the child’s age if she or he had survived to be enumerated by the census.

4 The Urban Transition Historical GIS organised by John Logan and Brown University is creating historical GIS maps of cities in the period 1880–1940 linked to complete-count census data. Currently, these maps have focused on northern cities (Shertzer et al., 2016) and enumeration district maps for the 1930 and 1940 censuses. For details on the project, see https://s4.ad.brown.edu/Projects/UTP2/index.htm.

5 We also used EDs to construct traditional aspatial city-level measures of racial evenness and isolation (the dissimilarity and isolation indexes, *DI* and *II*) and used the measures in analyses of the urban population. We limited our analysis here to the relationship between child mortality and segregation at the ED level for several reasons. First, the dissimilarity and isolation indexes suggested relatively low levels of urban segregation—Blacks and Whites could be found in large numbers in nearly every ED of most southern cities—despite high levels of within neighbourhood segregation (Grigoryeva & Ruef, 2015). Aspatial city-level measures, in other words, masked significant heterogeneity within subunits. Second, significant disparities in the local disease environment rendered intercity comparisons risky. As discussed below, in our analysis of the relationship between child mortality and segregation at the ED level we employed county-level fixed effects to control for unobserved heterogeneity in the disease environment. The small number of southern cities in most counties in the early 20th-century South, however, precluded the use of county-level fixed effects in models using the city-level measures *DI* and *II*. In the online Appendix S1, we show the results of regression models using *DI* and *II* and state-level fixed effects models. Although the results of these models appear reasonable—all else being equal a higher *DI* or *II* was typically associated with modestly higher child mortality among Black children and little difference among White children—we deemed these results subject to greater bias. In addition, unlike the ED-level variables *proportion Black* and *SIS*, the addition of *DI* and *II* resulted in the degradation of the models. Models with *DI* and *II* had larger black-white differentials in mortality relative to the baseline models than models with *proportion Black* and *SIS* alone.

6 The expected number of runs is defined as *E*(*R*) = (2 × *N*_1_*N*_2_/(*N*_1_ + *N*_2_)) + 1, where *N*_1_ and *N*_2_ represents the number of households from the two racial groups in the ED. Negative values are possible but rare (Grigoryeva & Ruef, 2015).

7 Although we know that each child born and dying in the 5 years preceding each census died before the enumeration—and therefore died before reaching age 5—we do not know their exact year of death or age at death, preventing the use of event history analyses.
