# Supplementary material for: Racial residential segregation and child mortality in the southern United States at the turn of the 20th century
Source: Popul Space Place. Author manuscript; Available in PMC 2024 Nov 15. (PMC11566120; doi:10.1002/psp.2678)
Supplement: appendix [file NIHMS1981795-supplement-appendix.pdf]

Supplementary Appendix

J'Mag Karbeah and J. David Hacker
June 1, 2023

Comparison of Results Using Reconstructed Birth Histories and the Child Mortality Index

As noted in the main text, we considered several measures of child mortality used by other investigators, including whether a mother had experienced the death of one or more of her children (Logan and Parman 2018), the proportion of a mother's children who were deceased at the time of the census, and a child mortality index standardized to a model life table (Conner 2017; Dribe *et al.* 2020; Hacker and Haines 2005; Haines and Preston 1997; Preston and Haines 1991; Preston *et al.* 1994; Reid *et al.* 2016). The mortality index is in some ways ideal. It performs well in regression models, is easy to interpret, and unlike the proportion of a mother's children dying or whether a mother had experienced the death of one or more of their children, is not biased by differences in women's age, marital duration, and number of children ever born. As detailed elsewhere (Haines and Preston 1997; United Nations 1983), the mortality index is constructed by dividing the actual number of a mother's children dying by the *expected* number of children dying. The expected number varies by the number of a mother's children ever born, the model life table chosen as a standard, and an estimate of the number of years that the mother's children experienced at risk of death. The estimated number of at-risk years experienced by a mother's children is derived from the mother's age or martial duration and parity progression ratios by age or marital duration among all ever-married women. It is often presented in the form of a "mortality reference period," the average number of years prior to the census that a mother's children were at risk of death. If mortality was changing over time—as it was in the early twentieth century, when mortality rates were declining—the mortality reference period can be included as an independent variable in models of child mortality to control for temporal trends in the risk of dying (e.g., Dribe et al. 2020).

Although easy to construct, the mortality index and mortality reference period measures are

made at the mother level and do not attempt to estimate the date specific children were born or, if a child was deceased, their approximate date or age at death. Because an individual's place of residence and the level of racial residential segregation they experienced were time-dependent, we considered this to be a serious limitation. The deceased child of a 45-year old mother in the 1910 census, for example, may have been born as early as about 1880, when the mother was aged 15, or as late as April 15, 1910 (the nominal date of the 1910 census), when the mother was aged 45. His or her age at death may have ranged from a low of less than one day to a high of more than 30 years. During this possible three-decade interval, the mother and child may have lived in different places with different levels of residential segregation than observed in the 1910 census. Even among non-migrants, city and neighborhood segregation patterns may have changed over time.

Because reconstructed birth histories rely on more information (each mother's age, the ages of her coresident children and length of the intervals between each child, age-specific fertility rates of women in her birth cohort, age-specific mortality rates for children by birth cohort, age-specific rates of children leaving home, and other characteristics of the mother and her spouse) compared to the mortality reference date estimation (which relies only on mothers' ages or marriage durations and parity-progression ratios by age among women in mothers' birth cohorts), the result is a better estimate of when children were born, when they were exposed to the risk of death, and when they died (Hacker 2020). When reconstructed birth histories are limited to births occurring in the five years preceding each census, at-risk children and their parents can be assumed to have been more likely to have experienced the conditions observed at the time of the census relative to older children and their parents. In addition, all children in the analysis can be assumed to have died before the age of 5. Although it is possible to use the mortality reference date variable in a similar way (e.g., by defining the analytical population as mothers with a child mortality reference date less than five years prior to the census), the reference date is the *average* year at risk experienced among a mother's children. It cannot be used to

precisely define which children were at risk during the years immediately prior to the census and which children were not at risk. Reconstructed birth histories allow us to impose these selection criteria and thereby greatly reduce, but not eliminate, unobserved migration and potential endogeneity between segregation and mortality.

Although the imputation procedure is probabilistic and subject to uncertainties, the overall number of child deaths experienced by mothers are unaffected; the reconstruction procedure is used only to establish the likely timing of deceased children's births. Comparison of the results to own-child fertility estimates indicates that birth reconstructions are extremely accurate at the aggregate level, yielding age-specific fertility rates nearly identical to cohort estimates, mortality differentials nearly identical to differentials estimated with the more commonly-used mortality index, and mortality trends consistent with expectations.

In Table A1, we show the results of our analysis when using the child mortality index instead of live births from mothers' reconstructed birth histories. Following Dribe et al. (2020), we constructed the child mortality index using Model West level 13.5 as our standard and mothers' ages and parity progression ratios by age (unfortunately, the IPUMS 1900 and 1910 complete-count datasets do not include the times married variable, precluding the use of marital durations and parity progression ratios by marital duration). To make the comparison as close as possible, we limited the analytical population to mothers with a mortality reference date within five years of each census. In practice, this means the analysis is limited to mothers aged 15-29 at the time of the census. Although the analysis may include children aged 15 years or more with ages at death of up to 15 years (children born to mothers in their upper 20s at the time of the census who gave birth in their early teenage years), the typical child represented in the analysis was born in the five years prior to each census and died before the age of five. We used ordinary least square regression, weighted by the number of children ever born, and county-level fixed effects models with standard errors clustered at the census enumeration district.

The results largely conform with the results shown in Tables 4 and 5. Once again, we find that Black children were more likely to die than White children, even after controlling for all variables, and that the risk of death among Black children relative to White children was much higher in urban areas. Among the variables of interest, *proportion Black* was positively correlated with Black and White child mortality in all models as it also was in Tables 4 and 5. And once again, the results indicate that the *SIS* was positively correlated with the risk of death among Black children only in rural models for 1900 and 1910 and in the urban models for 1910.

Comparison of Results Under an Assumption of Differential Census Underestimation of Children by Race

Estimates of census coverage errors by age and sex for the native-born white population suggest that significant numbers of children—particularly those aged 0 and 1 at the time of the census—were undercounted in the 1900 and 1910 censuses (Hacker 2013). No estimates of census coverage errors by have been made for the Black population prior to 1930, when the net undercount was estimated to be about 14.1% for Black children aged 0-4 and 5.5% for Black children aged 5-9 (Preston et al. 2003). Age distributions of the Black and native-born White populations, however, strongly suggests that Black infants and young children were also more likely to be missed by the 1900 and 1910 censuses relative to White infants and young children. A very rough estimate of the *net* census undercount of children aged 0-1 relative to older children—the net effect of children being missed by the census entirely or incorrectly enumerated with an older age—can be made by back projecting the number of White and Black births in each of the ten years prior to each census using life table survival estimates and assuming constant population growth over the decade. The results for the 1910 census suggest a net undercount of 25.9% among Black children aged 0-1 compared to a net undercount of 6.7% among White children of the same age. If we were to inflate the number of living children by these crude undercount estimates—

or those made by Preston et al. for the 1930 census—estimated race differential in child mortality would be reduced—about 10-15 percent in most models—but remain large.

In addition to biasing the overall race differential in child mortality, census under-enumeration differentials by age and race may bias our probabilistic assignment of ages/years of birth for mothers' "unmatched" children (children who were living at the time of the census but not present in their mothers' households). Ordinarily, the imputation procedure for unmatched children is based on probabilities derived from observed patterns of coresidence and non-coresidence of mothers and their own children by single years of age, adjusted in high-mortality contexts for maternal mortality (Luther and Cho 1988). For 1900 and 1910, our estimates indicated that less than one percent of children under the age of 15 *who had living mothers at the time of the census* lived in different households than their mothers. Increasingly after age 15, however, mothers and their own children lived in different households. At age 21, we estimated that 41.3% of White children and 50.8% of Black children with living mothers were no longer coresident with their mothers at the time of the census. In these cases, the number of surviving children reported by the mother will exceed the number of own children observed coresiding with the mother in the household. Because the ages of unmatched children are not observed, they need to be imputed. Given the age patterns of non-coresidence observed in the cross-section as an input, our imputation program is much more likely assign an unmatched child an age above 15 than an age below 15, provided the mother was old enough to have a 15-year-old child.[1] A 40-year old mother who had one child ever born, one child surviving, but no children coresident with her in the census, for example, was much more likely to have had her child 15-19 years prior to the census, when she was aged 20-24, than 0-4 years prior to the census, when she was aged 35-39, and therefore the unmatched child will more likely be assigned an age above age 15 than an age below age 5 by the

[1] The actual probability for an unmatched child at each age is established by the joint probability the child was living apart from his or her mother and the corresponding age-specific fertility rate for women in the mother's birth cohort.

imputation routine.

If a significant percentage of children were under-counted by the census, however, the probability that an unmatched child was an infant or a younger child increases significantly. Given the large race differentials in census under-enumeration suggested by our (admittedly crude) estimates, our imputation procedures may be assigning too few Black children aged 0-1 relative to White children aged 0-1 and our regression results could be biased. We therefore decided to test the sensitivity of our results to the assumption that younger Black children were more likely to be missed by the census. We did so by modifying the assumed age pattern of unmatched children to yield the number of White and Black children aged 0 and 1 suggested by our crude undercount estimates, using those probabilities to construct a revised version of the dataset, and repeating our analysis.

Table A2 shows the regression results corresponding to the bottom panels of Tables 4 and 5 (the models for combined rural and urban areas) in the main text using the dataset adjusted for assumed race differentials in census under-enumeration. (We obtained similar results in separate rural and urban models). As expected, the race differential in child mortality was reduced significantly in models based on the adjusted dataset. Among children born to mothers in urban areas in 1910, for example, the coefficient for Black children in the models without segregation variables fell from 1.405 in Table 5 using the unadjusted dataset (40.5% higher mortality among Black children, all else being equal) to 1.289 using the dataset adjusted for probably under-enumeration (28.9% higher mortality). The results for our segregation variables, however, remain largely the same, with similar sized coefficients and similar changes in the race differential in child mortality explained by the segregation variables relative to the baseline models. These results confirm that our results are robust to potentially large race differentials in census under-enumeration.

Results using City-Level Measures of Segregation

Aspatial dissimilarity and isolation indexes can be constructed with historical census data using cities as the macro-unit and enumeration districts (EDs) as the micro-units for urban areas with more than one ED (Cutler and Glaeser 1997; White and Borrell 2011). Although aspatial city-level measures have been criticized for their failure to account for the spatial patterning of sub-units and possible masking of significant heterogeneity within subunits, they are commonly used. We therefore constructed both measures and included them is analyses of child mortality in the urban population.

The formula for Dissimilarity Index (*DI*) was:

$$DI = \frac{1}{2}\sum_{i=1}^{N}\left|\frac{w_i}{W} - \frac{b_i}{B}\right|$$

where w_i is the number of households headed by White individuals in the ith enumeration district of a city, W is the total number of White-headed households of the city, b_i is the number of households headed by Black individuals in the ith enumeration district, and B is the total number of Black-headed households in the city. The dissimilarity Index can be thought of as the proportion of a city's household heads who must move across enumeration district boundaries to obtain the same race distributions in each ED.

Using the same notation, the formula for the Isolation Index (*II*) was:

$$II = \sum_{i=1}^{N}\left(\frac{b_i}{b_i + w_i} * \frac{b_i}{B}\right)$$

The Isolation Index measures the degree of potential contact, or lack of contact, between Black and White people in each city. It can be thought of as the degree to which the average Black household is isolated from White households.

In Table A3, we show the results of our analysis showing baseline regression results with no segregation variables (Model 1 for 1900 and Model 5 for 1910), models with *proportion Black* and the

SIS (Models 2 and 6), models with the *DI* and an interaction variable for children's race added (Models 3 and 7), and models with the *II* and an interaction variable added (Models 4 and 8). Because most southern counties in 1900 included one or fewer urban places, the models use state-level fixed effects instead of county-level fixed effects. Results, therefore, are subject to greater potential biases from unobserved heterogeneities than the models shown in Tables 4 and 5 of the main text. Focusing on the new segregation variables, the *DI* and *II* were not correlated with the mortality of White children in urban areas of the South in either census year. The mortality of Black children, however, was moderately higher in cities with higher dissimilarity indexes in both census years, indicating that an uneven distribution of Blacks and Whites across southern cities was associated with lower child survival. The mortality of Black children was also higher in southern cities in 1910 with higher isolation indexes, indicating the greater isolation of Blacks and Whites was correlated with worse outcomes for Black children. The interaction was also positive in 1900, but not statistically significant. It is interesting to note that while both variables were associated with the mortality of Black children in the expected direction, the unexplained differential between the mortality of White and Black children, which fell 17.2% in 1900 between models 1 and 2 and 20.8% in 1910 between models 5 and 6 with the addition of *proportion Black*, *SIS*, and their associated interaction variables, was lower after the addition of the *DI*, the *II*, and their associated interaction variables.

Additional citations not included in the main text

J. David Hacker, J. D. 2013. New estimates of census coverage in the United States, 1850-1930, *Social Science History*, 37, 71-101.

Preston, S. H., I. T. Elo, M. E. Hill, and I. Rosenwaike. 2003. *The Demography of African Americans, 1930-1990*. Springer.

Table A1. OLS regression models of child mortality index, southern census regions, 1900 and 1910 censuses

	Rural Areas		Urban Areas	
Census year	1900	1910	1900	1910
Model number	(1)	(2)	(3)	(4)
Characteristics of child				
Black children	0.253 ***	0.268 ***	0.595 ***	0.594 ***
Mortality period	-0.032 ***	-0.053 ***	-0.030 ***	-0.033 ***
Characteristics of mother				
In paid labor force	0.086 ***	0.086 ***	0.310 ***	0.341 ***
Characteristics of father				
Farmer	-0.191 ***	-0.182 ***	-0.160 ***	-0.073
Professional or technical	-0.153 ***	-0.121 ***	-0.252 ***	-0.153 ***
Managers, Officials, Prop.	-0.132 ***	-0.116 ***	-0.181 ***	-0.124 ***
Sales and clerks	-0.147 ***	-0.191 ***	-0.245 ***	-0.153 ***
Craftsmen	-0.049 **	-0.052 ***	-0.044 **	-0.009
Apprentices, operatives	0.051 **	0.081 ***	-0.002	0.046 **
Service worker	-0.046	0.047 ***	-0.107 ***	-0.050 *
Farm Laborer	-0.091 ***	-0.061 ***	-0.007	-0.084
No occupation or non-valid	-0.142 ***	-0.049 ***	-0.153 ***	0.089
Currently unemployed		-0.009		0.108 ***
Unemployed 1-52 weeks		0.055 ***		0.097 ***
Characteristics of parents				
Homeowners	-0.063 ***	-0.062 ***	-0.105 ***	-0.093 ***
Both parents literate	-0.153 ***	-0.158 ***	-0.283 ***	-0.313 ***
Residence characteristics				
Urban city, pop.>25,000			-0.024	-0.044
Segregation Measures				
Proportion Black in ED (*prop. Black*)	0.038 ***	0.015 ***	0.121 ***	-0.072 **
Child's race Black * *prop. Black*	0.388 ***	0.267 ***	0.247 ***	0.425 **
Seq. index of segreg. (*SIS*)	-0.029	-0.015	0.005	0.014
Child's race Black * *SIS*	0.242 ***	0.168 ***	-0.200	0.152 ***
Constant	1.331 ***	1.377 ***	1.481 ***	1.324 ***
Observations (weighted)	3,307,981	3,812,352	659,743	1,220,857
Fixed effects unit	County	County	County	County
Number of enumeration districts	9,515	11,516	2,245	3,608
Number of counties	1,108	1,179	325	454
R-square	0.037	0.034	0.069	0.070
Mean child mortality index	1.129	1.129	1.320	1.194
Mean child mortality index, Whites	0.936	0.940	0.988	0.872
Mean child mortality index, Blacks	1.402	1.407	1.911	1.804

Notes: County-level fixed effects OLS regression of the child mortality index weighted by children ever born with standard errors clusted at the enumeration district level. The mortality index was constructed using mothers' ages and standardized on the Model West level 13.5 life table (see Dribe et al. 2020 for details). The analytical dataset includes mothers aged 15-29 years in the 1900 and 1910 IPUMS complete-counted datasets who were: (1) enumerated with valid children ever born and children surviving data; (2) enumerated in a residence location that could be clearly identified as urban or rural; (3) currently married at the time of the census; (4) living in a southern census region; (5) in marriages having a duration of five or more years; and (6) living in the same household as their husbands. Reference categories are mothers who were not in the paid labor force; could not read and write or was married to a husband who could not read or write; lived (in urban models only) in a city with 25,000 or fewer inhabitants; rented their homes; and whose husbands were employed as general laborers. *** p<0.001, ** p<0.01, * p<0.05

Table A2. Comparision of logistic regression models of child mortality in reconstructed birth histories adjusted for assumed race differentials in census enumeration of children aged 0 and 1.

	1900 - unadjusted				1900 - adjusted			
Model	Baseline		Full		Baseline		Full	
Child's race								
White	*ref.*		*ref.*		*ref.*		*ref.*	
Black	1.330	***	1.258	***	1.289	***	1.231	***
Segregation Measures								
Proportion Black in ED			1.192	***			1.114	***
Child's race Black * Prop. Black			1.352	***			1.253	***
Seq. index of segreg. (*SIS*)			0.962				0.965	*
Child's race Black * *SIS*			1.473	***			1.320	***
Observations	2,212,524		2,212,524		2,525,905		2,525,905	
Pseudo r-square	0.047		0.047		0.039		0.039	
Change in race differential			21.8%				20.1%	

	1910 - unadjusted				1910 - adjusted			
Model	Baseline		Full		Baseline		Full	
Child's race								
White	*ref.*		*ref.*		*ref.*		*ref.*	
Black	1.405	***	1.333	***	1.289	***	1.231	***
Segregation Measures								
Proportion Black in ED			1.122	***			1.114	***
Child's race Black * Prop. Black			1.314	***			1.253	***
Seq. index of segreg. (*SIS*)			0.955	**			0.965	*
Child's race Black * *SIS*			1.354	***			1.320	***
Observations	2,464,691		2,464,691		2,525,905		2,525,905	
Pseudo r-square	0.050		0.050		0.039		0.039	
Change in race differential			17.8%				20.1%	

Notes: See Table 4. "Unadjusted" model results for 1900 and 1910 are based on observed and imputed births in women's reconstructed birth histories in the 1900 and 1910 censuses. Births for "unmatched" children were obtained using observed age patterns of coresidence between mothers and their own children by age and race. If effect, the imputation process assumes no race differentials in census coverage by age. Results for the 1900 and 1910 "adjusted" models are based on an alternative version of women's reconstructed birth histories. Births for unmatched children were obtained using age patterns of coresidence adjusted to obtain about 25.9% more Black children and 6.7% more White children aged 0-1 to evaluate the robustness of the model results to probable race differentials in census coverage.

*** p<0.001, ** p<0.01, * p<0.05

Table A3. Logistic regression models of child mortality, urban areas in southern census regions, 1900 and 1910 censuses

Census year	1900				1910			
Model number	(1)	(2)	(3)	(4)	(5)	(6)	(7)	(8)
Characteristics of child								
Black	1.558 ***	1.462 ***	1.470 ***	1.469 ***	1.606 ***	1.480 ***	1.483 ***	1.484 ***
Birth order	1.163 ***	1.163 ***	1.163 ***	1.163 ***	1.162 ***	1.162 ***	1.162 ***	1.162 ***
Years born prior to census (0-4)	1.065 ***	1.065 ***	1.065 ***	1.065 ***	1.068 ***	1.068 ***	1.068 ***	1.068 ***
Characteristics of mother								
Age at birth of child	0.991 ***	0.991 ***	0.991 ***	0.991 ***	0.994 **	0.994 **	0.994 **	0.994 **
In paid labor force	1.263 ***	1.257 ***	1.254 ***	1.254 ***	1.293 ***	1.293 ***	1.293 ***	1.293 ***
Characteristics of father								
Farmer	0.810 ***	0.807 ***	0.812 ***	0.808 ***	0.828 ***	0.828 ***	0.834 ***	0.832 ***
Professional or technical	0.870 ***	0.868 ***	0.870 ***	0.869 ***	0.847 ***	0.846 ***	0.847 ***	0.847 ***
Managers, Officials, Prop.	0.889 ***	0.885 ***	0.885 ***	0.885 ***	0.925 ***	0.927 ***	0.926 ***	0.926 ***
Sales and clerks	0.886 ***	0.884 ***	0.884 ***	0.884 ***	0.867 ***	0.868 ***	0.868 ***	0.868 ***
Craftsmen	0.963 *	0.959 *	0.960 *	0.960 *	0.992	0.990	0.991	0.991
Apprentices, operatives	0.996	0.996	0.995	0.996	1.023	1.028	1.027	1.027
Service worker	0.979	0.974	0.970	0.971	1.054 **	1.054 **	1.052 **	1.053 **
Farm Laborer	0.857 ***	0.854 ***	0.871 ***	0.865 ***	0.884 **	0.890 **	0.902 **	0.900 **
No occupation or non-valid	0.913 ***	0.915 ***	0.919 ***	0.917 ***	1.216 ***	1.221 ***	1.221 ***	1.221 ***
Currently unemployed					1.087 ***	1.087 ***	1.086 ***	1.087 ***
Unemployed 1-52 weeks					1.063 ***	1.068 ***	1.067 ***	1.068 ***
Characteristics of parents								
Homeowners	0.906 ***	0.904 ***	0.907 ***	0.906 ***	0.901 ***	0.900 ***	0.903 ***	0.903 ***
Both parents literate	0.882 ***	0.882 ***	0.880 ***	0.880 ***	0.858 ***	0.856 ***	0.854 ***	0.854 ***
Residence characteristics								
Urban city, pop.>25,000	1.120 ***	1.111 ***	1.038	1.069 *	1.148 ***	1.134 ***	1.100 **	1.107 **
Segregation Measures								
Proportion Black in ED		1.155 *	1.133 *	1.137 *		0.981	0.968	0.970
Child's race Black * *Prop. Black*		1.281 **	1.28 **	1.258 *		1.404 ***	1.391 ***	1.376 ***
Seq. index of segreg. (*SIS*)		0.927	0.940	0.936		1.066	1.069	1.071
Child's race Black * *SIS*		1.105	1.092	1.091		1.200 *	1.205 *	1.193 *
Dissmiliarity index (*DI*)			1.141				1.017	
Child's race Black * *DI*			1.256 **				1.191 **	
Isolation index (*II*)				1.114				1.007
Child's race Black * *II*				1.368				1.290 *
Observations	356,119	356,119	356,119	356,119	471,542	471,542	471,542	471,542
Fixed effects unit	State	State	State	State	State	State	State	State
Number of urban places	344	344	344	344	487	487	487	487
Number of states	17	17	17	17	17	17	17	17
Pseudo r-square	0.051	0.052	0.052	0.052	0.055	0.055	0.055	0.055

Change In race differential	17.2%	15.8%	15.9%		20.8%	20.3%	20.1%

Notes: See Table 1 in the main text for selection criteria. Reference categories are White children born to mothers who were not in the paid labor force; whose fathers were employed as general laborers; whose parents rented their homes, whose parents included one or more partners who could not read and write, and--in urban models--lived in a city with 25,000 or fewer inhabitants. *** $p<0.001$, ** $p<0.01$, * $p<0.05$
